# LIM and SH3 protein 1 regulates cell growth and chemosensitivity of human glioblastoma via the PI3K/AKT pathway

**DOI:** 10.1186/s12885-018-4649-2

**Published:** 2018-07-06

**Authors:** Chuanhong Zhong, Yitian Chen, Bei Tao, Lilei Peng, Tangming Peng, Xiaobo Yang, Xiangguo Xia, Ligang Chen

**Affiliations:** 1 Neurosurgery Department, Affiliated Hospital of Southwest Medical University, Luzhou, 646000 China; 2 Department of Clinical Medicine, Medical College of Soochow University, Suzhou, China; 3 Rheumatism Department, Affiliated Hospital of Southwest Medical University, Luzhou, China

**Keywords:** LIM and SH3 protein 1, Glioblastoma, Temozolomide, PI3K/AKT pathway

## Abstract

**Background:**

LIM and SH3 protein 1 (LASP1) is upregulated in several types of human cancer and implicated in cancer progression. However, the expression and intrinsic function of LASP1 in glioblastoma (GBM) remains unclear.

**Method:**

Oncomine and The Cancer Genome Atlas (TCGA) database was analyzed for the expression and clinical significance of LASP1 in GBM. LASP1 mRNA and protein level were measured by qRT-PCR and western blotting. The effect of LASP1 on GBM proliferation was examined by MTT assay and colony formation assay, the effect of LASP1 on sensitivity of Temozolomide was measured by flow cytometry and subcutaneous tumor model. The association between LASP1 and PI3K/AKT signaling was assessed by western blotting.

**Results:**

Oncomine GBM dataset analysis indicated LASP1 is significantly upregulated in GBM tissues compared to normal tissues. GBM dataset from The Cancer Genome Atlas (TCGA) revealed that high LASP1 expression is related to poor overall survival. LASP1 mRNA and protein in clinical specimens and tumor cell lines are frequently overexpressed. LASP1 knockdown dramatically suppressed U87 and U251 cell proliferation. Silencing LASP1 potentiated cell chemosensitivity to temozolomide in vitro, LASP1 knockdown inhibited tumor growth and enhanced the therapeutic effect of temozolomide in vivo. TCGA dataset analysis indicated LASP1 was correlated with PI3K/AKT signaling pathway, and LASP1 deletion inhibited this pathway. Combination treatment with PI3K/AKT pathway inhibitor LY294002 dramatically accelerated the suppression effect of temozolomide.

**Conclusion:**

LASP1 may function as an oncogene in GBM and regulate cell proliferation and chemosensitivity in a PI3K/AKT-dependent mechanism. Thus, the LASP1/PI3K/AKT axis is a promising target and therapeutic strategy for GBM treatment.

## Background

Glioblastoma (GBM) is the most aggressive type of brain tumor and originates in the parenchyma. The first-line therapeutic strategy is maximal surgery resection, followed by radiotherapy combined with temozolomide (TMZ) chemotherapy [1]. However, most patients will die within 2 years [2]. The poor survival is due to multiple factors, including excessive proliferation of glioblastoma cells and chemoresistance to temozolomide or radioresistance [3]. Thus, it is urgent to identify key molecules in proliferation and chemoresistance, which may serve as potential drugs targets and improve the survival of glioblastoma patients.

Recently, LIM and SH3 domain-containing proteins were reported to be upregulated in tumors and associated with a wide spectrum of cellular processes such as proliferation, migration, tumorigenesis, and chemoresistance [4, 5]. LIM and SH3 protein 1(LASP1) is a member of the nebulin protein family and contains both LIM and SH3 domains [6]. High LASP1 expression has been detected in breast cancer [7, 8], colorectal cancer [9–12], pancreatic cancer [13], and prostate cancer [8]. Moreover, bioinformatics analysis showed LASP1 is upregulated in glioblastoma and related to poor overall survival, but the complex function and molecular mechanism of LASP1 in GBM remains largely unknown.

PI3K families are lipid kinases involved in multiple fundamental process including proliferation, cell metabolism, and tumorigenesis [14]. According to substrate specificity and subsequence homology analysis, PI3K is classified into three classes. Class I shows the strongest relationship with cancer. Class I contains a catalytic subunit p110 (α, β, γ) and the regulator subunit p85. When p110 is activated, it can catalyze the conversion of phosphatidy-linositol-3, 4-bisphosphate to the second messenger phosphatidylinositol-3, 4, 5-bisphosphate, which binds the pleckstrin homology domain of AKT and phosphorylates Thr308 and Ser473. Activated AKT mediates various downstream substrates and promotes cell survival [14]. Similarly, activation of the PI3K/AKT pathway in GBM leads to cell proliferation and TMZ drug resistance [15]. Previous studies reported that LASP1 activates the PI3K/AKT pathway in colorectal cancer [11, 16] and gallbladder cancer [17]; notably, The Cancer Genome Atlas (TCGA) dataset analysis showed LASP1 was positively related to the PI3K/AKT pathway. Based on previous research and bioinformatics analysis, we predicted that LASP1 is a vital modulator of the PI3K/AKT pathway and mediates GBM proliferation and therapy resistance.

In the present study, we first investigated the expression pattern and molecular function of LASP1 in GBM and found that LASP1 is required for GBM proliferation and reduced the chemotherapy sensitivity of TMZ both in vitro and in vivo. Mechanically, LASP1 activated the PI3K/AKT pathway. These findings improve the understanding of LASP1 in influencing GBM proliferation, TMZ resistance, and PI3K/AKT signaling pathway. Our data indicate that LASP1 is a potential therapeutic target in GBM.

## Methods

### Clinical patient tissues

Fresh primary GBM tissues and paired normal brain tissues were collected from 38 patients at the Affiliated Hospital of Southwest Medical University. Diagnosis of each primary GBM was confirmed by experienced pathologists. No patients received treatment prior to operation. Written informed consent was acquired from the patients for research purposes. This study was approved and supervised by The Ethics Committee of Southwest Medical University and all aspects of this study comply with the Declaration of Helsinki.

### Cell culture

GBM cell lines LN229(CA NO.CRL-2611), U251(CA NO.TCHu-58), U87(CA NO.HTB-14), and T98G(CA NO.CRL-1690) were obtained from the Cell Bank of the Chinese Academy of Sciences (Shanghai, China) and ATCC, USA. LN229, U87, and T98G cells were cultured in Eagle’s Minimum Essential Medium (MEM; Hyclone, Logan, UT, USA) and U251 cells were cultured in Dulbecco’s modified Eagle’s medium (DMEM; Hyclone). All media were supplemented with 10% fetal bovine serum (Thermo Scientific, Waltham, MA, USA). The cells were cultured at 37 °C with a humidity of 90–95 and 5% CO_2_. All cells were propagated for less than 6 months after resuscitation.

### siRNA and stable knockdown construction

After propagation, cells were transfected with 100 nM Lipofectamine 2000 reagent (Invitrogen, Carlsbad, CA, USA). LASP1-specific siRNA was purchased from GenePharma (Shanghai, China). The siRNA sequences were as follows:

siRNA1 5′-CGCGCGGUGUAUGACUAAdTdT-3′;

siRNA2 5′-GAAUCAACAAGACCCAGGAdTdT-3′;

siRNA3 5′-CGCGCGGUGUACUAGACUAdTdT-3′;

Negative control siRNA 5′-TTCTCCGAACGTGTCACGT-3′.

Based on their knockdown efficiencies, siRNA3 was selected to synthesized shRNA by GenePharma (Shanghai, China). Transfection of shRNA was performed according to the manufacturer’s instructions. After 1 week of transfection, cells were selected with 1 μg/mL puromycin. Knockdown efficiency was confirmed by western blotting.

### cDNA synthesis and quantitative real-time PCR

Total RNA was extracted by using a Trizol kit according to the manufacturer’s instructions. After extraction, 1 μg total RNA was reverse-transcribed by using Takara RT reagent according to the manufacturer’s protocol. Each cDNA was subjected to quantitative real-time PCR in triplicate on a LightCycler 480 system (Roche, Basel, Switzerland). The following primers were used in this study: GAPDH (F: ACCCAGAAGACTGTGGATGG, R: TCTAGACGGCAGGTCAGGTC), LASP1 (F: ATGAACCCCAACTGCGCC, R: TCAGATGGCCTCCACGTAGTT).

### MTT assays

For proliferation analysis, 1000 cells were plated in 5 replicates in 96-well plates after gene transfection. For TMZ sensitivity analysis, 5000 cells were seeded in 96-well plates. After culturing the cells for the indicated times, the media were replaced with 3-(4,5-dimethylthiazol-2-yl)-2,5-diphenyltetrazolium bromide (MTT) followed by co-culture for 4 h, and then 150 μL DMSO was added before measured at 570 nm in a multimode plate reader (Perkin Elmer, Waltham, MA, USA) according to the manufacturer’s instructions.

### Western blotting (WB)

Cells were washed with 5% PBS 3 times and then lysed in RIPA lysis buffer for 1 h. Protein quantification was performed using BCA kits. Equal amounts of proteins were separated by SDS-PAGE and transferred onto polyvinylidene difluoride membranes (Amersham Pharmacia Biotech, Amersham, UK). The proteins were labeled with specific antibodies against LASP1 (1:1000; Chemicon, Temecula, CA, USA); cleaved poly (ADP-ribose) polymerase (PARP), cleaved caspase3, caspase3,p-PI3K, PI3K, p-Akt(Ser473), Akt (1:1000, Cell Signaling Technology, Danvers, MA, USA); and GAPDH (1:1000, Santa Cruz Biotechnology, Dallas, TX, USA). Autoradiography signals were quantified and analyzed using Quantity One software (Bio-Rad, Hercules, CA, USA).

### Colony formation and EdU assays

For colony formation assays, 1000 cells were plated in 6-well plates at 2 mL/well and the medium was replaced every 3 days. After 2 weeks, colonies were fixed with 4% paraformaldehyde, stained with 0.1% crystal violet and analyzed.

For 5-ethynyl-2′-deoxyuridine (EdU) experiments, cells were transferred into a 96-well plate at 2000 cells/well; 24 h later, cells were dyed with EdU Cell Proliferation Assay Kit (RiboBio, Guangzhou, China) according to the standard protocol, and photographed with a fluorescence microscope (Olympus, Tokyo, Japan).

### Xenograft tumor growth

Male nude mice were maintained in a barrier facility and this study was approved by the Ethical Committee of Southwest Medical University. All procedures were conducted according to approved protocols. Stable shRNA against LASP1 cells and control cells (1 × 10^6^ cells in 150 μL PBS) were injected into the right subcutaneous space of nude mice. For the TMZ therapy group, at 1 week, mice were administered TMZ by oral gavage at a concentration of 100 μM per day for 5 days per week, with therapy continuing for 3 weeks. One month after injection, mice were sacrificed, tumors were dissected, and tumor volumes were measured as follows: length × width^2^ × ½. Tumor weight was measured using a scale.

### Statistical analysis

Data were analyzed by SPSS version 19.0 software (SPSS, Chicago, IL, USA). Each experiment was performed at least three times independently and values are shown as the mean ± standard deviation (SD). Two-tailed Student’s *t*-tests were used to estimate the significance between two independent groups. For the quantitative reverse transcription (qRT)-PCR assay, if the distribution of the data was abnormal, the Wilcoxon rank-sum test and Kruskal–Wallis test were used, while the Student’s *t*-test was used for normally distributed data.

## Results

### LASP1 is up-regulated in GBM

**Fig. 1:**
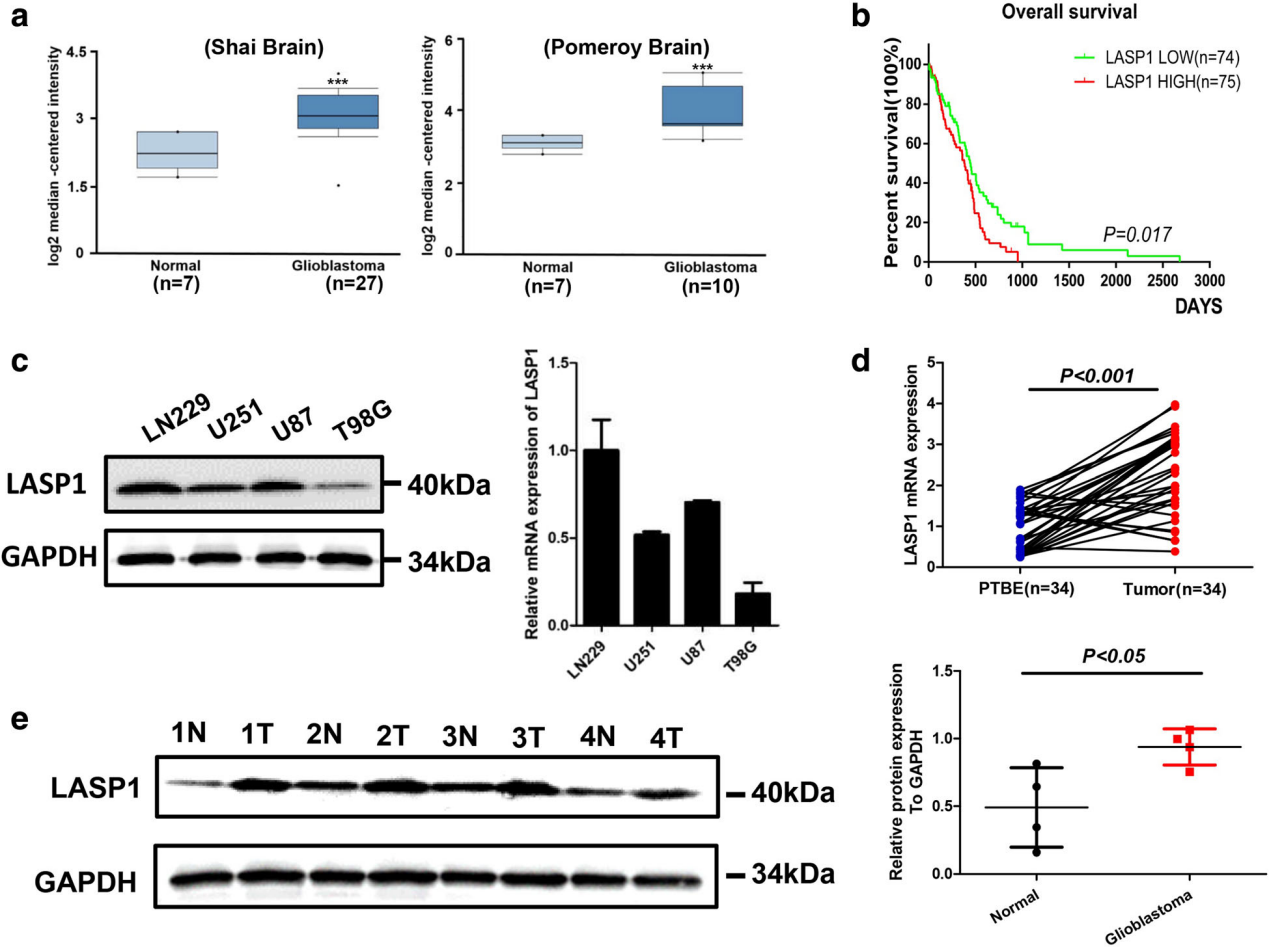
ASP1 is up-regulated in GBM. **a** Oncomine analysis of LASP1 expression in two independent GBM databases; (**b**) Kaplan-Meier survival analysis of 149 GBM patients from TCGA; patients were divided into a high group (*n* = 75) and low group (*n* = 74) according to LASP1 expression; (**c**) western blot and real-time PCR analysis were performed to detect LASP1 expression in 4 GBM cell lines; (**d**) real-time PCR analysis of LASP1 mRNA expression in paired human GBM tissues (*n* = 34) and their adjacent normal tissues (n = 34); (**e**) western blot analysis of LASP1 expression in 4 paired samples of GBM tissues versus adjacent normal tissues. The immunosignal was quantified by greyscale scanning software Quantity One, and the relative protein abundance was normalized by GAPDH. ^*^ *P < 0.05,* ^**^ *P < 0.01,* ^***^p < 0.001

To investigate the role of LASP1 in GBM, we analyzed the Oncomine database. In two independent GBM databases (Fig. 1a), LASP1 was upregulated in tumor samples compared to in normal tissues (*P < 0.001*). Moreover, Kaplan-Meier analysis of TCGA dataset for GBM revealed that patients with high expression of LASP1 had worse overall survival (OS) compared to the LASP1 low expression group (*P = 0.017*; Fig. 1b). These data suggest that LASP1 is an oncogene in GBM and correlated with tumor development. Consistently, western blot was used to detect the protein level of LASP1 and qRT-PCR was conducted to detect the mRNA level of LASP1 in 4 GBM cells (Fig. 1c). According to the results, U87 and U251 cells with moderate expression were used in further experiments. The expression of LASP1 mRNA in GBM tissues was measured by qRT-PCR, and high expression of LASP1 mRNA was detected in 27 of 35 GBM samples compared to in control samples (Fig. 1d, *P < 0.001*). Furthermore, high expression of LASP1 protein was also observed in GBM samples compared to in paired normal tissues (Fig. 1e*, P = 0.03*).

### Inhibition of LASP1 suppresses GBM cell proliferation in vitro

**Fig. 2:**
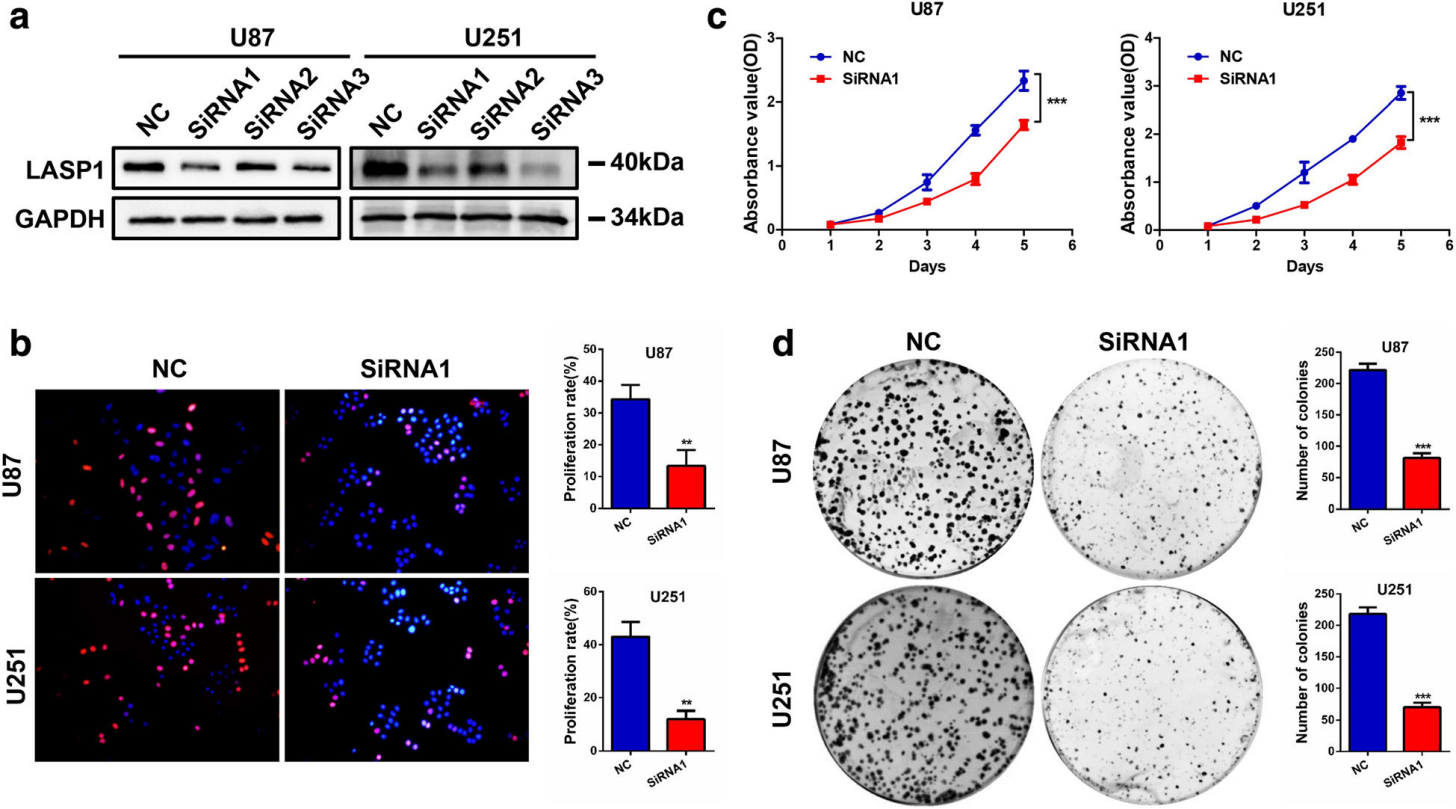
Inhibition of LASP1 suppresses GBM cell proliferation. **a** Transfection rate of LASP1 siRNAs was detected by western blotting; EdU incorporation assays (**b**), MTT assays (**c**), and colony formation assays (**d**) were performed after transfection with NC and si-LASP1. Scale bar: 50 μm. Error bars show the mean ± standard deviation. For 3 independent experiments. ^*^ *P < 0.05,* ^**^ *P < 0.01.* ^***^p < 0.001

To explore the biological function of LASP1 in GBM, we transfected 3 siRNAs against LASP1 in U87 and U251 cells. In both cells, siRNA-1 showed the most effective suppression rate and was chosen for the following experiment (Fig. 2a). EdU incorporation assays showed LASP1 inhibition reduced proliferation rates in both U87 and U251 cells (Fig. 2b). Additionally, MTT assays and colony formation assays showed that LASP1 silencing lead to weaker cell viability and decreased the number of colonies (Fig. 2c, d).

### Inhibition of LASP1 promotes sensitivity of TMZ in vitro

**Fig. 3:**
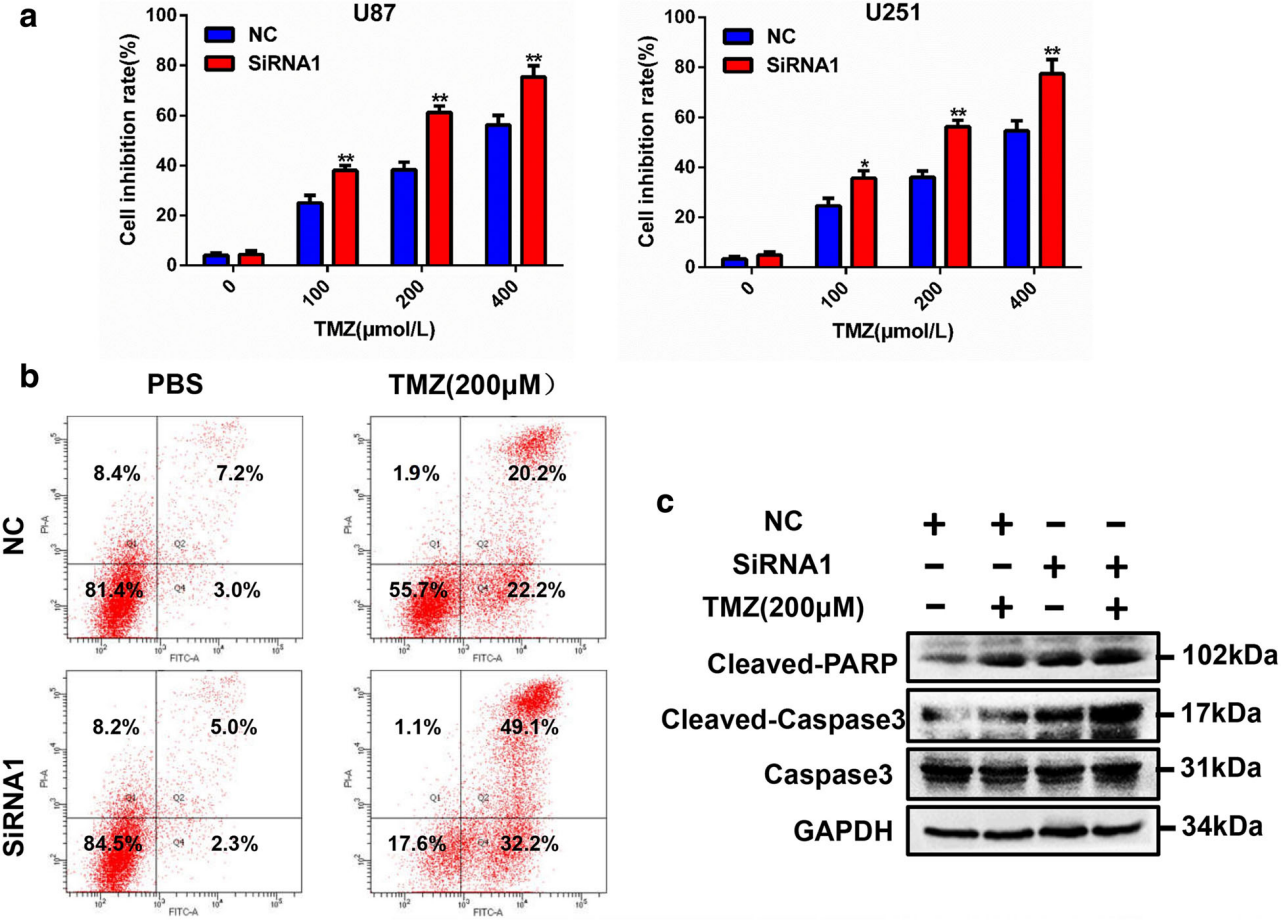
Inhibition of LASP1 promotes sensitivity of TMZ. **a** LASP1 expression significantly changed cell sensitivity to TMZ in U87 and U251 cells; (**b**) u87 cells were transfected LASP1 siRNA and flow cytometry was used for apoptosis analysis after transfection; (**c**) changes in the expression of cleaved PARP, cleaved caspase 3, and caspase 3 in response to LASP-1 alteration as measured by western blotting. GAPDH served as a loading control. ^*^ *P < 0.05,* ^**^ *P < 0.01.* ^***^p < 0.001

Temozolomide is the most commonly used drug for GBM. To investigate the potential effect of LASP1 on TMZ therapy, we performed MTT assays by incubating U87 and U251 cells with different concentrations of TMZ for 24 h, and then calculated the inhibition rates of the cells. Notably, following suppression of LASP1, the inhibition rates induced by TMZ were dramatically increased compared to in the NC groups (Fig. 3a). U87 cells were treated with 200 μM TMZ for 24 h, and then flow cytometry was used to measure the apoptosis rate. The results showed that silencing of LASP1 increased the apoptosis rate, particularly when combined with TMZ treatment (Fig. 3b). We further measured the protein expression of apoptosis markers such as cleaved PARP and cleaved caspase 3, under combined treated with 200 μM after LASP1 silencing. These two apoptosis indicators were increased by more than in the control group (Fig. 3c), indicating that silencing of LASP1 improved chemotherapy sensitivity and promoted apoptosis of TMZ-treated GBM cells. Thus, LASP1 may be a sensitivity indicator of TMZ therapy and an attractive target for overcoming TMZ chemoresistance.

### LASP1 knockdown reduces tumor growth and promotes chemosensitivity in vivo

**Fig. 4:**
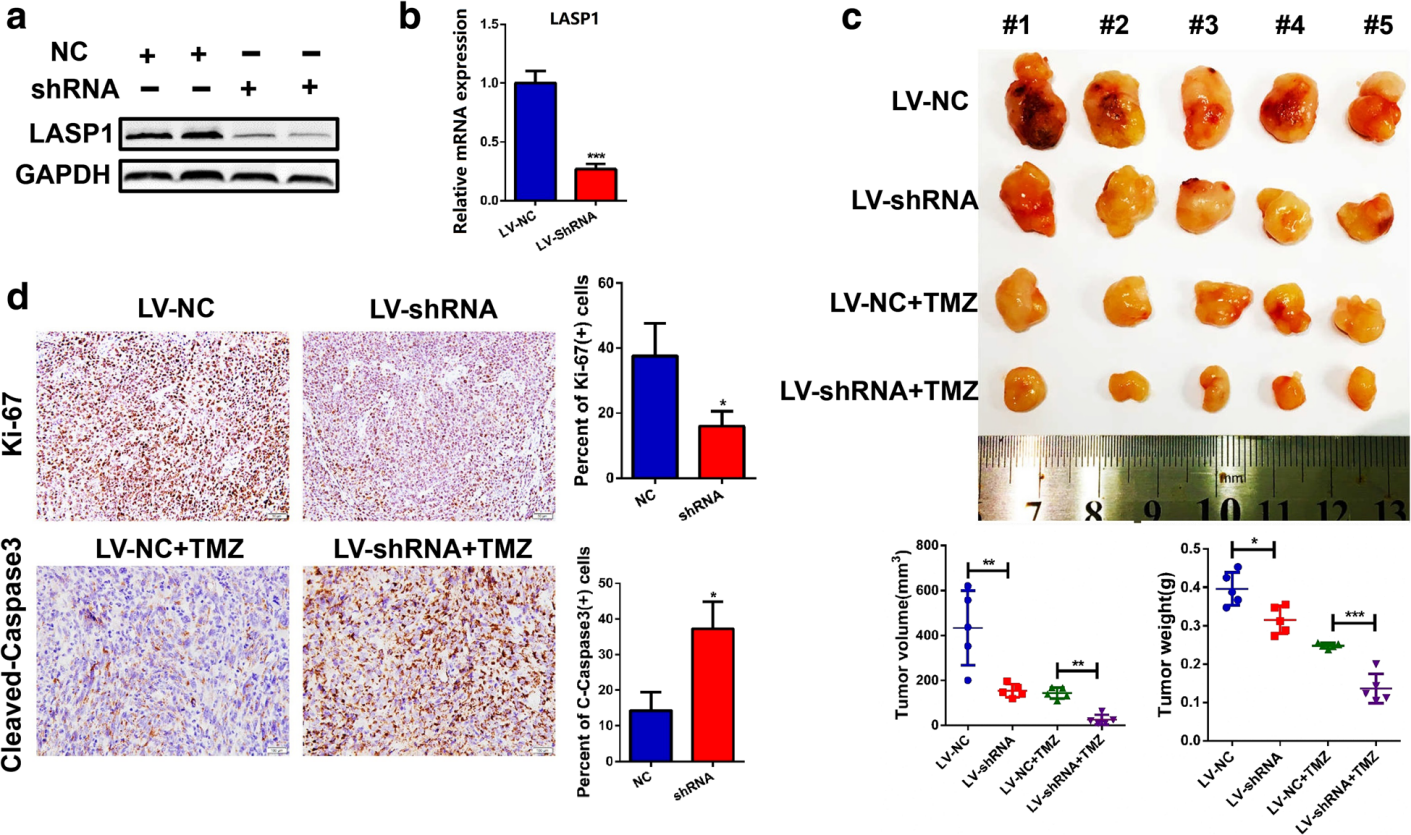
LASP1 knockdown reduces tumor growth and promotes chemosensitivity in vivo. **a** U87 cells were transfected with lentivirus-based shRNA against LASP1, and western blotting was performed to determine knockdown efficiency; (**b**) qRT-PCR assay showed inhibition of LASP1 at the mRNA level; (**c**) stable construction tumor cells were injected subcutaneously into the back of nude mice to detect proliferative ability and chemosensitivity to TMZ. Tumor volume and weight were measured at 30 days after injection and expressed as the mean ± SD, *n* = 5; (**d**) proliferative ability of tumor cells was measured by Ki-67 and apoptosis rate was derived by cleaved caspase3 immunohistochemical staining, Scale bar, 50 μm. ^*^ *P < 0.05,* ^**^ *P < 0.01.* ^***^p < 0.001

To further evaluate the biological function of LASP1 in GBM, a lentivirus-based shRNA was synthesized and U87 cells were used for stable knockdown construction. Western blotting and qRT-PCR assays confirmed remarkable suppression of LASP1 expression in LV-shRNA cells (Fig. 4a, b). In vivo, a subcutaneous tumor model was used to assess the effect of LASP1 on tumor growth and chemosensitivity of TMZ. As shown in Fig. 4c, LV-shRNA group cells grew more slowly than control cells. Following TMZ treatment, tumor nodules in the LV-shRNA group showed the smallest volumes and lowest weights. Similarly, immunohistochemistry staining of Ki-67 showed a lower positive index in the LV-shRNA group compared to in the control. In addition, following TMZ treatment, LV-shRNA group cells showed high cleaved caspase3 staining (Fig. 4d), indicating that knockdown of LASP1 induced high levels of apoptosis following TMZ treatment.

### LASP1 regulates pivotal biologic process by activating PI3K/AKT pathway

**Fig. 5:**
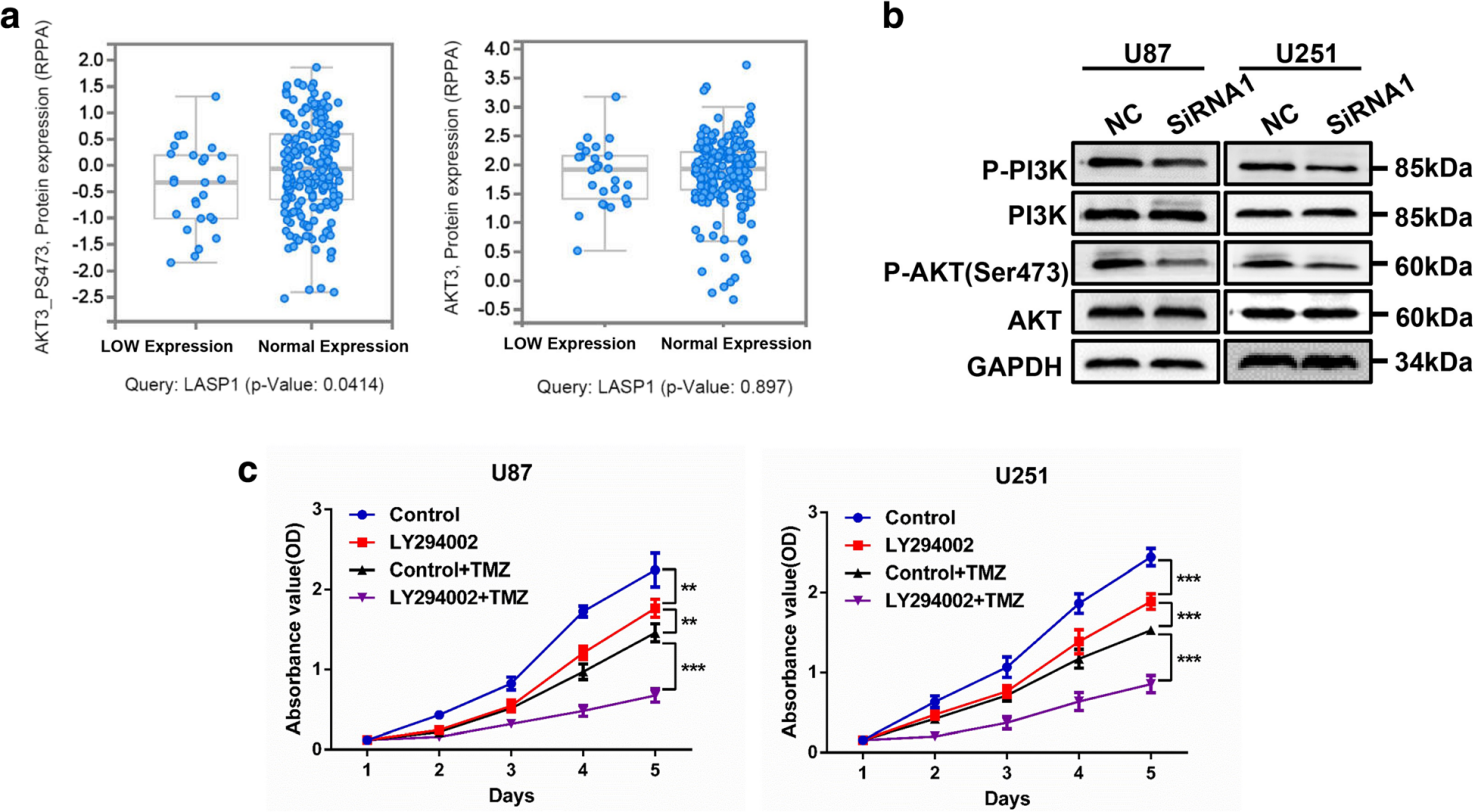
LASP1 regulated pivotal biologic process by activating PI3K/AKT pathway. **a** Glioblastoma data in TCGA database was analyzed by using cBioPortal tools (http://www.cbioportal.org/index.do). Each blue dot indicates one case of glioblastoma. Phosphorylated AKT3 at Ser473 is positively correlated with LASP1 mRNA level, while the total level of AKT3 has no significant correlation with LASP1; (**b**) Western blotting analysis of p-PI3K, PI3K, p-AKT(Ser473), AKT proteins in indicated cells transfected with si-LASP1; (**c**) LY294002 inhibited tumor growth and dramatically accelerated the suppression effect of TMZ in U87 and U251 cell lines. ^*^ *P < 0.05,* ^**^ *P < 0.01.* ^***^p < 0.001

To determine the underlying mechanism of LASP1 in regulating proliferation and TMZ chemoresistance, we analyzed glioblastoma data in TCGA database (TCGA, Cell 2013) by using cBioPortal tools (http://www.cbioportal.org/index.do) and found that phosphorylated AKT3 at Ser 473 was positively correlated with the LASP1 mRNA level, but the total level of AKT3 was not significantly correlated with LASP 1 (Fig. 5a). These results indicate that LASP1 positively regulates the PI3K/AKT pathway, which plays an essential role in regulating proliferation and chemotherapy sensitivity [15, 18]. Western blotting verified with the knockdown of LASP1 expression, and the phosphorylation level of PI3K and AKT at Ser473 were clearly inhibited, while the total protein levels of PI3K and AKT were maintained (Fig. 5b).

Furthermore, the association between PI3K/AKT signaling and GBM was confirmed using the PI3K/AKT pathway inhibitor LY294002. Treatment with LY294002 markedly suppressed U87 and U251 cells growth compared with control group. Notably, in combination treated with TMZ (50 μM), LY294002 dramatically accelerated the suppression effect of TMZ in both cell lines (Fig. 5c). In conclusion, we demonstrated that LASP1 was upregulated in GBM and positively correlated with cell proliferation ability and resistance to TMZ therapy, and these effects may partly activate the PI3K/AKT pathway.

## Discussion

GBMs, the most common and aggressive central nervous system tumors, exhibit poor prognosis because of excessive growth of tumor cells and treatment resistance, particularly secondary resistance to TMZ therapy [2, 19]. Although some signaling pathways such as the PI3K/AKT pathway have been reported to be involved in cell proliferation and TMZ treatment failure [15], the underlying mechanisms of these pathways are unclear.

LASP1, an actin-binding protein, has a LIM cysteine-rich domain at its N-terminus and SRC homology region 3 (SH3) domain at its C-terminus. Through these structures, LASP1 can interact with other structures and signaling proteins [5]. In GBM, the biological function of LASP1 has never been characterized. According to bioinformatics analysis of the Oncomine and TCGA databases, we found LASP1 was also upregulated in GBM and associated with an unfavorable prognosis (Fig. 1a, b). In this study, we analyzed the mRNA and protein expression of LASP1 in fresh GBM tissues and paired normal tissues (Fig. 1c–e). Our data clearly show that LASP1 was overexpressed in GBM, and thus LASP1 may be involved in the carcinogenesis of GBM. Functionally, LASP1 was correlated with cell proliferation rate and colony formation ability (Fig. 2b–d), while silencing of LASP1 markedly enhanced chemosensitivity of TMZ. To further analyze these results, we determined the effect of LASP1 on the cell apoptosis rate by flow cytometry. As expected, depletion of LASP1 accelerated the apoptosis rate induced by TMZ, as well as influenced the apoptosis markers (Fig. 3b, c). Moreover, a subcutaneous tumor model confirmed that LASP1 was strongly associated with the tumor growth and therapy effect of TMZ (Fig. 4c, d). These functional assays suggest an oncogenic role for LASP1 in GBM development and chemoresistance.

LASP1 was initially identified from a cDNA library of metastatic axillary lymph nodes in breast cancer [6], suggesting that it acts as a tumor metastasis-associated protein in cancer. Recent studies identified LASP1 as an oncogenic gene in various types of cancer and showed that LASP1 strongly promoted the migration, invasion, and epithelial-mesenchymal transition abilities of cancer cells [7–13] . However, LASP1 was not confined to metastasis and LASP1 also affects cancer proliferation and may be associated with the drug response. For example, LASP1 promoted cell growth in CRC cells and induced cell cycle arrested in the S and G2/M phases [9]. miR-1 and miR-133a, which inhibited LASP1 expression by directly binding to its 3′ untranslated region, decreased cell proliferation rates in vivo and in vitro [16, 20]. Similar results have also been observed in gallbladder cancer [17], prostate cancer [21], and hepatocellular carcinoma [22]. Notably, Li et al. showed that LINC00672, a long non-coding RNA that recruits hnRNPs to suppress the expression of LASP1, increased the chemosensitivity of paclitaxel in endometrial cancer, indicating that LASP1 impacts the drug response in cancer.

Generally, hyper-activation of signaling pathways including the PI3K/AKT pathway has frequently been observed in cancers and plays a central role in regulating cell survival, proliferation, metastasis, angiogenesis, metabolism, and chemoresistance [15, 23]. To explore the signaling downstream of LASP1, we analyzed TCGA dataset of stomach adenocarcinoma and found that the level of AKT phosphorylated at Ser473 was positively correlated with the expression of LASP1 mRNA (Fig. 5a). Additionally, LASP1 has been reported as an upstream mediator of the PI3K/AKT pathway. In colorectal cancer, Zhao et al. performed proteomic assays and found that LASP1 interacted with 14–3-3σ and decreased the expression of 14–3-3σ, as 14–3-3σ could also interact with AKT and suppress AKT phosphorylation, the deletion of 14–3-3σ contributed to LASP1-mediated activation of the PI3K/AKT pathway [11, 16]. In addition, LASP1 reportedly promoted S100P expression via the PI3K/AKT pathway and induced proliferation, metastasis, and cell cycle arrest at the G2/M phase [17]. Therefore, we predicted that LASP1 mediates the PI3K/AKT pathway in GBM and is involved in cell proliferation and chemoresistance. In this study, We confirmed that LASP1 increased the phosphorylation level of PI3K and AKT (Fig. 5b), treated GBM cells with PI3K/AKT pathway inhibitor LY294002 suppressed tumor growth and enhance the chemosensitivity of TMZ (Fig. 5c). Based on our results and those of previous studies, we confirmed that LASP1 is an essential mediator of the PI3K/AKT pathway and that activation of PI3K/AKT is involved in regulating GBM proliferation and TMZ resistance.

## Conclusion

In summary, we verified that LASP1 is upregulated in GBM and promotes GBM cell proliferation and TMZ resistance by activating the PI3K/AKT pathway. These findings provide insight into the oncogenic role of LASP1 in GBM and highlight the potential of LASP1 in the anticancer therapy of GBM patients.
